# Animal studies reveal that the ghrelin pathway regulates alcohol-mediated responses

**DOI:** 10.3389/fpsyt.2023.1050973

**Published:** 2023-03-08

**Authors:** Elisabet Jerlhag

**Affiliations:** Department of Pharmacology, Institute of Neuroscience and Physiology, The Sahlgrenska Academy at the University of Gothenburg, Gothenburg, Sweden

**Keywords:** appetite-regulatory peptides, gut-brain axis, alcohol, addictive drugs, dopamine, reward, addiction, dependence

## Abstract

Alcohol use disorder (AUD) is often described as repeated phases of binge drinking, compulsive alcohol-taking, craving for alcohol during withdrawal, and drinking with an aim to a reduce the negative consequences. Although multifaceted, alcohol-induced reward is one aspect influencing the former three of these. The neurobiological mechanisms regulating AUD processes are complex and one of these systems is the gut-brain peptide ghrelin. The vast physiological properties of ghrelin are mediated *via* growth hormone secretagogue receptor (GHSR, ghrelin receptor). Ghrelin is well known for its ability to control feeding, hunger, and metabolism. Moreover, ghrelin signaling appears central for alcohol-mediated responses; findings reviewed herein. In male rodents GHSR antagonism reduces alcohol consumption, prevents relapse drinking, and attenuates the motivation to consume alcohol. On the other hand, ghrelin increases the consumption of alcohol. This ghrelin-alcohol interaction is also verified to some extent in humans with high alcohol consumption. In addition, either pharmacological or genetic suppression of GHSR decreases several alcohol-related effects (behavioral or neurochemical). Indeed, this suppression blocks the alcohol-induced hyperlocomotion and dopamine release in nucleus accumbens as well as ablates the alcohol reward in the conditioned place preference model. Although not fully elucidated, this interaction appears to involve areas central for reward, such as the ventral tegmental area (VTA) and brain nodes targeted by VTA projections. As reviewed briefly, the ghrelin pathway does not only modulate alcohol-mediated effects, it regulates reward-related behaviors induced by addictive drugs. Although personality traits like impulsivity and risk-taking behaviors are common in patients with AUD, the role of the ghrelin pathway thereof is unknown and remains to be studied. In summary, the ghrelin pathway regulates addiction processes like AUD and therefore the possibility that GHSR antagonism reduces alcohol or drug-taking should be explored in randomized clinical trials.

## 1. Introduction

In society harmful alcohol consumption is a global health problem, where alcohol use disorder (AUD) is a top pathology associated with this risky consumption (1). The health problems and socioeconomic burden of AUD for individuals and society in large are major concerns (2, 3). AUD is often referred to a cycle of different phases that are repeated over time [for review see Koob (4)]. Alcohol-induced reward is one central aspect influencing phases like the initial binge phase, the latter compulsive alcohol-taking and craving for alcohol during withdrawal. Besides these, drinking with an aim to a reduce the negative consequences is another important phase. An AUD diagnosis is defined according to a set classification system which includes parameters such as loss of intake control, persistent desire, craving and tolerance. Although not a part of the AUD diagnosis, both risk taking and impulsivity are common characteristics observed in patients with AUD (5). A complex disease like AUD involves a vast number of underlying pathways and neurobiological substrates which are being identified by means of both preclinical and clinical research. During latter years gut-brain peptides appear to be important players for the AUD process, possibly due to an interference with alcohol-induced reward [for review see Jerlhag (6)]. To date, multiple gut-brain peptides exist and ghrelin is one of these.

The stomach-derived hormone ghrelin (acyl-ghrelin) exerts its physiological effects *via* activation of growth hormone secretagogue receptor (GHSR), a receptor known for its intrinsic activity and ability to heterodimer with other receptors [for review see Cornejo et al. (7)]. The receptor is today often referred to the ghrelin receptor, and earned its initial name from the growth hormone releasing ability after activation of the receptor (8). Ghrelin is to date the only identified orexigenic (appetite promoting) gut-brain peptide (8). Notably, the feeding aspects influenced by ghrelin include both the hedonic and homeostatic (7, 9). The homeostatic properties of ghrelin are further evident as it promotes hunger, appetite and body weight gain [for review see Cornejo et al. (7)]. Ghrelin also has a myriad of other physiological properties as it for instance regulates secretion of gastric acid and gastric motility [for review see Cornejo et al. (7)]. This review summarizes the available research on if and how the ghrelin pathway modulates alcohol-related effects (behaviors and neurochemistry) in animals. It should be noted that there are no clinically available GHSR ligands, but for research purposes antagonists like JMV2959 and [D-Lys^3^]-GHRP-6 and the inverse agonist PF-5190457 are used. The name of these have been omitted throughout the review to increase the readability of the review. First, the ability of ghrelin signaling to modulate different aspects of alcohol drinking is introduced. Then the review will cover the effect of genetic or pharmacological suppression of its receptor on the rewarding aspects of alcohol. Further, this reward reduction is suggested as a tentative explanation to why the alcohol consumption is reduced after GHSR suppression. Thereafter, brain regions and neurocircuits central for the ghrelin-alcohol interaction will be introduced. The review will then briefly cover the role of ghrelin pathway in drug taking. Moreover, confounding factors are described. On a final note, future directions are discussed and the clinical trial testing the effect of GHSR antagonists/inverse agonists on alcohol drinking in AUD patients is suggested as a concluding remark.

The reviewed articles presented herein were selected by means of keywords and the included articles were quality checked before inclusion (Table 1). As this is a review rather than systematic overview, the presented articles were thus not selected using PICOS, PRISM, Cochrane, or JBI. A systematic overview provides a higher reliability, and the lack of such design should be considered as a limitation. It should also be noted that previous reviews cover aspects of this research field. In these, several gut-rain peptides are introduced, the clinical aspects/data or alcohol-associated liver disease are introduced in detail [examples of other reviews within the field (10–14)]. The novelty of this review is the detail insight into available preclinical studies, where the link between ghrelin-pathway and alcohol-related effects is described in detail. Moreover, novel future directions are discussed.

**TABLE 1:** Main keys words for the selected articles presented in the present review.

Main key words for ghrelin pathway	Ghrelin, acyl-ghrelin, des-acyl-ghrelin, GOAT, JMV2959, GHSR, GHSR-1A, ghrelin receptor
Main key words for alcohol and addiction	Alcohol, ethanol, alcohol use disorder, alcohol addiction, alcohol dependence, reward, dopamine, addictive drugs, mesocorticolimbic dopamine system, nucleus accumbens, ventral tegmental area
Main key words for methods	Alcohol intake, consumption, alcohol preference, alcohol-seeking, operant self-administration, motivation to consume alcohol, relapse drinking, locomotor activity, condition place preference, dopamine release, progressive ratio
Additional key words	Cocaine, amphetamine, methamphetamine, cannabinoids, LSD, opioids, ecstasy, nicotine, addictive drugs, drugs of abuse

Article search was conducted 30th of August, 20th of September, and 6th of December 2022. No exclusion criteria were used. Quality check was based on scientific experience and refers to quality of the methods, number of animals included, design of experiments.

## 2. The role of the ghrelin pathway on alcohol-related effects in animals

### 2.1. Inhibition of the ghrelin pathway reduces various alcohol drinking behaviors in rodents

**FIGURE 1:**
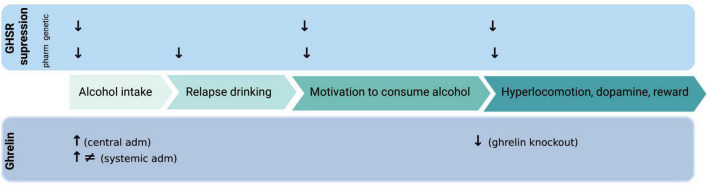
Illustrative summary of available preclinical studies on how ghrelin administration (adm) or genetic/pharmacologic (pharm) GHSR (growth hormone receptor, ghrelin receptor) suppression influences various alcohol-related responses. ↓, decreases; ↑, increases; ≠, no effect. Created with BioRender.com.

The ability of GHSR antagonists to reduce alcohol drinking has been shown in numerous preclinical studies (Figure 1). It was initially found that acute administration of a GHSR antagonist lowers alcohol intake in male mice (15), high alcohol preferring male rats (16) or male rats consuming alcohol for long periods of time (17). Notably, a dose-dependent reduction was evident after repeated injections of a GHSR antagonist (17). The findings that the decline in alcohol drinking is greater at seven compared to three months of alcohol exposure (17) are interesting from clinical perspective. Indeed, it is plausible that a GHSR antagonist would reduce alcohol drinking more in patients with severe AUD compared to those with a mild AUD. The lack of tolerance toward the GHSR antagonist in drinking experiments (17) is further clinically beneficial, as it indicates that there would not be a dose adjustment during treatment. Although these initial findings report that GHSR antagonists are effective in rodents consuming alcohol for extensive periods of time prior to treatment, the ability of an antagonist to reduce drinking is also evident in animals consuming alcohol for shorter periods of time. On this note, male mice/rats/prairie voles exposed to alcohol shortly before treatment display a decline in alcohol consumption after acute or repeated treatment with a GHSR antagonist (18–22). The ability of GHSR to regulate alcohol consumption is further evident as alcohol drinking is lower in GHSR knockout male mice (15) or rats (23) compared to wild-type littermates. From a mechanistic perspective, vagal afferents appear to be involved in the ability of a GHSR antagonist to reduce alcohol intake in male rats (24).

In support for these initial findings where a decline in alcohol drinking after GHSR inhibition is reported, are additional studies demonstrating a beneficial role of GHSR antagonists on other alcohol drinking behaviors. One of these is relapse drinking after abstinence, a trait blocked by a GHSR antagonist in male mice (15) and male rats (17). These findings may be intriguing from a clinical perspective as a declined relapse drinking may reflect a reduction in alcohol craving, which is observed in humans during relapse. Moreover, pharmacological (16, 19) or genetic (23) suppression of the GHSR attenuates the motivation to consume alcohol in the operant self-administration paradigm in male rats. As for regular alcohol intake, the ability of a GHSR antagonist to reduce self-administration is persistent in animals exposed to alcohol for long or short periods of time before treatment (16, 19, 23). Moreover, this reduction is also observed in female rats treated with a GHSR antagonist into the nucleus accumbens (NAc) (25). Together these findings indicate that GHSR regulate aspects of the AUD process such as escalated intake, craving for alcohol and motivation to consume alcohol. A clinical relevance is provided as polymorphisms of the GHSR gene is associated with high alcohol intake or AUD diagnosis (26–28). Moreover, in patients with AUD an inverse GHSR-1A agonist reduces self-reported alcohol craving (29) as well as decreases hangover symptoms after intravenous self-administration of alcohol (30). Collectively, these studies imply that GHSR are required for various alcohol drinking behaviors. However, individuals drink alcohol due to various reasons where impulsivity and risk-taking are important features. Upcoming studies should therefore explore the role of the ghrelin pathway for such characteristics in patients with AUD.

Opposed to the clear outcome on alcohol drinking by the GHSR antagonists administered into the brain or systemically, the role of central versus peripheral ghrelin is unclear. An initial study reveals that male mice with an overall ghrelin knockout drink less alcohol compared to their wild-type littermates (18). Moreover, ghrelin administration into the ventricles of the brain increases alcohol intake in male mice consuming alcohol for months before the ghrelin infusion (15). An effect also observed after a systemic administration in one study of male rats (31), but not in another study of male mice (32) exposed to alcohol for a short-period of time. Moreover, neutralization of circulating ghrelin does not influence alcohol intake in male rats drinking alcohol for months prior to treatment (33). The reasons for these diverging results remain to be determined, but may be due to parameters such as different drinking models, time of alcohol exposure prior to test or animals/strain used. The role of circulating ghrelin for AUD processes is further discussed as circulating levels of ghrelin are reduced by acute alcohol in male rats (34, 35) and enhanced by alcohol drinking (36). Whereas another study reveals no differences in plasma ghrelin between low and high alcohol-preferring rats (16). The association between ghrelin and alcohol in humans is extensively reviewed elsewhere (6). In brief, in patients with AUD the high plasma levels of ghrelin is associated with the craving for alcohol (37–41), possibly involving the ventral striatum (41). An area also central for ghrelin-alcohol interaction in animals (25). On the same note, in patients with AUD intravenous infusion of a high dose of ghrelin declines the latency to first alcohol containing drink (42) and elevates alcohol craving in patients with AUD (43). Besides, polymorphisms in the *pre*-pro-ghrelin gene are associated with aspects of the AUD diagnosis (28, 44, 45).

Collectively, these findings raise the discussion whether circulating or central ghrelin signaling is required for a modulation of the alcohol-related effects, an aspect that should be addressed in upcoming studies. It should also be noted that the divergence between central and peripheral signaling exist when it comes to another gut-brain peptide; glucagon-like peptide-1 (GLP-1). Indeed, central, but not peripheral GLP-1 receptors appear to be important for the ability of a GLP-1 receptor agonist to attenuate alcohol-related responses in male mice (46).

### 2.2. Suppression of the ghrelin pathway attenuates the alcohol-related responses (behavioral or neurochemical) in rodents

The decline in alcohol drinking by GHSR antagonists in animals exposed to long or short-term alcohol may be due to a reduction in alcohol’s rewarding experience as this also is reduced by GHSR antagonism (Figure 1). A feature essential for the several phase of the addiction cycle (4) and associated to the development of AUD later in life (47). In animal models alcohol reward can be measured by its ability to activate the mesolimbic dopamine system, consisting of dopamine projections from the ventral tegmental area (VTA) to the NAc shell [for review see Jayaram-Lindström et al. (48)]. Specifically, alcohol causes a locomotor stimulation, dopamine release in NAc shell and causes a conditioned place preference (CPP) in rodents that are alcohol naïve prior to test [for review see Jayaram-Lindstroöm et al. (48) and Sanchis-Segura and Spanagel (49)]. Importantly, the elevated dopamine in NAc shell is translated into human studies, where the dopamine release is positively correlated to the reward of alcohol [for review see Jayaram-Lindström et al. (48)]. It should however be noted that this correlation is influenced by factors such as sex (50), possibly due to alcohol’s ability to affect multiple neurotransmitters. When it comes to ghrelin pathway and alcohol reward, preclinical studies of male mice reveal that the ability of alcohol to cause a locomotor stimulation, dopamine release in NAc or CPP is blocked both by pharmacological or genetic suppression of the GHSR (15). A similar finding also observed after sub-chronic administration of a GHSR antagonist (51). On a similar note, these behavioral and neurochemical responses to alcohol are ablated in male mice with an overall ghrelin knockout (52). A finding later replicated as ghrelin knockout male mice do not display a CPP to alcohol (18). In mice using an overall ghrelin knockout the role of central versus peripheral ghrelin cannot be elucidated. In attempts to explore this, a study explored the alcohol responses after neutralization of circulating ghrelin (33). Intriguingly, neutralization of circulating ghrelin did not affect the alcohol-induced locomotor stimulation, dopamine release in NAc and CPP (33). As reviewed extensively elsewhere (10, 11, 13, 14), in humans (and rodents to some extent) associations between alcohol responses and plasma ghrelin have been found. Briefly, a high subjective intensity of alcohol is associated to circulating ghrelin levels (53). On a similar note, in a study with individuals self-administrating alcohol intravenously an intense subjective alcohol experience is positively associated with higher fasting ghrelin levels (54). Taken together, these studies imply that GHSR are required for alcohol to elicit reward and that the role of central versus peripheral ghrelin should be explored in detail.

### 2.3. Brain areas participating in the interaction between ghrelin and alcohol in rodents

**FIGURE 2:**
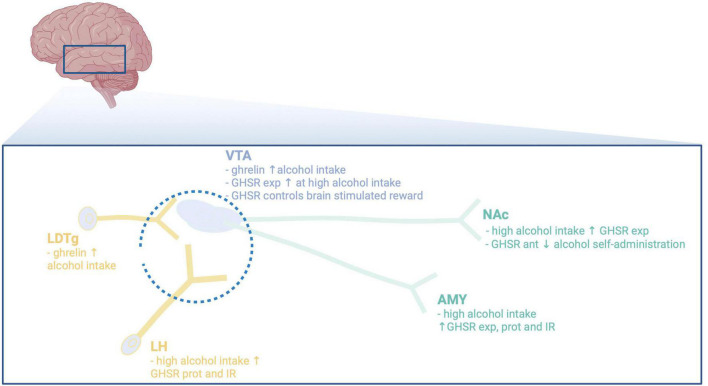
Illustrative summary of available preclinical studies on the main brain regions participating in the interaction between alcohol and the ghrelin pathway. VTA, ventral tegmental area; NAc, nucleus accumbens; LDTg, laterodorsal tegmental area; LH, lateral hypothalamus; AMY, amygdala; exp, expression; ant, antagonist; prot, protein; IR, immunoreactivity; ↓, decreases; ↑, increases. Created with BioRender.com.

Areas and circuits responsible for the ghrelin-alcohol interaction remain to be fully elucidated, but may involve areas central for reward (Figure 2). First of all, these brain regions express GHSR (55–57), indicating that these areas mediate ghrelin effects. When it comes to NAc, high alcohol intake elevates the GHSR expression in this area (17). Moreover, local infusion of a GHSR antagonist into the NAc blocks the operant self-administration of alcohol in female rats (25). A finding also apparent in humans, as the ability of ghrelin to cause craving for alcohol involves the ventral striatum (corresponding to NAc in rodents) (41). Furthermore, preclinical studies imply that GHSR within the VTA, an area interconnected with the NAc, may be central for the interaction of ghrelin pathway and alcohol. Initial studies demonstrate that local infusion of ghrelin into the VTA increases alcohol intake in male mice (15), and causes reward *per se* (51, 58–62). Similarly, male rats with short exposure to alcohol prior to treatment display elevated alcohol intake after ghrelin into the VTA (63). This interaction is further evident as high-alcohol consuming rats display higher expression levels of the GHSR in the VTA than low-alcohol consuming rats (17). However, this association was not replicated in a human study (64). Direct application of ghrelin into the VTA of male rodents increases the activity dopamine cells (65) and activates the mesolimbic dopamine system (59, 60, 65–68). Similarly, GHSR on dopamine neurons in the VTA controls brain stimulation reward induced by optogenetic stimulation (69). Within the VTA, a local dopamine release controlled by the GHSR possibly located on dopamine neurons (65) appears central for either ghrelin or alcohol to activate this reward pathway (68). On this note, the ability of GHSR to form a heterodimer with dopamine receptors regulate the activity of dopamine neurons (70–72). These findings collectively suggest that ghrelin and dopamine signaling interact within the VTA to control the ability of stimuli like alcohol to activate the mesolimbic dopamine system. NAc and VTA are bidirectionally interconnected and opposed to VTA-NAc dopamine projection the GABA projection from NAc to VTA does not regulate brain stimulation reward (69).

Besides NAc, the VTA projects to reward-related areas like amygdala [for review see Jayaram-Lindström et al. (48)], or to Edinger–Westphal nucleus (73). When it comes to the role of GHSR in the amygdala (56, 74), both preclinical and clinical studies reveal that GHSR within this area modulate the alcohol response. Specifically, ghrelin elevates the amygdala levels of GABA in alcohol dependent or naïve male rats (34, 74). Moreover, in this area the protein levels of GHSR and the immunoreactive GHSR cells are elevated by high alcohol intake (34), as are the GHSR expression (17). In humans, ghrelin enhances the ability of alcohol-related cues to activate amygdala an effect involving inflammation markers (42, 75). Although studied to less extent, a GHSR antagonist prevents the ability of alcohol to activate the perioculomotor urocortin-containing neurons of the Edinger–Westphal nucleus (20). The notion that the activity of dopaminergic neurons of the VTA is modulated by afferents from the laterodorsal tegmental area (LDTg) or lateral hypothalamus [for review see Larsson and Engel (76)], raises the possibility that these areas could participate in the ghrelin-alcohol link. Indeed, local infusion of ghrelin into the LDTg activates the dopaminergic neurons of the VTA and enhances alcohol drinking in male mice (15, 58). Additionally, alcohol elevates ghrelin and dopamine within the lateral hypothalamus in male rats with short or long-term alcohol exposure (34). On a similar note, high alcohol consumption increases the protein levels of GHSR and immunoreactive GHSR cells in lateral hypothalamus (34). As the expression of GHSR in the brain is wide spread (55–57), additional area and circuits most likely are involved and should be defined in more detail.

### 2.4. Antagonism of the ghrelin pathways decreases drug-related behaviors in rodents

In addition to alcohol-mediated responses, the ghrelin pathway appears to influence behaviors induced by addictive drugs such as the psychostimulants. Indeed, the locomotor stimulatory properties (77) and CPP reward (78, 79) of cocaine are enhanced by ghrelin in male rodents. An interaction plausibly involving the ghrelin pathway in the VTA (80, 81) and NAc (82). In contrast to ghrelin, systemic administration of a GHSR antagonist attenuates the cocaine-induced hyperlocomotion, NAc dopamine release and CPP in male mice (83–86). On a similar note, GHSR inhibition reduces the reinstatement of cocaine-seeking in rats with a history of cocaine taking (81). This is further supported as GHSR inhibition reduces the ability of cocaine to cause a locomotor sensitization (84, 85). These cocaine responses appear to be mediated *via* central rather than peripheral ghrelin (86). Although cocaine does not alter the plasma levels of ghrelin in humans (87), enhanced circulating ghrelin is associated with cocaine seeking and cocaine taking in male rats (88, 89). Likewise, in male rodents a GHSR antagonist reduces relapse and drug-seeking for methamphetamine (90) and decreases the amphetamine-induced hyperlocomotion, NAc-dopamine and CPP (51, 68, 83). On a similar note, GHSR antagonism prevents nicotine (91–94) to activate the mesolimbic dopamine system in male rodents. Moreover, ghrelin potentiates the nicotine-induced dopamine release in striatal rat brain slices (92). These interactions between addictive drugs and ghrelin pathway in rodents are supported by few human genetic studies, where polymorphism of the GHSR genes is associated with either amphetamine or nicotine dependence (28, 95). Although the associations between ghrelin and smoking in humans are contradictory (96–102), craving for nicotine during abstinence appear positively associated with plasma ghrelin (101, 103). A finding in line with the association between ghrelin and alcohol craving (37–41).

The opioid crisis is a world-wide health problem, and novel treatment options is thus needed for opioid addictions. Intriguingly, GHSR could be a potential target as antagonists suppress the reward aspects and the drug taking of various opioid including morphine and fentanyl in male rodents (104–108). Another opioid interacting with the ghrelin pathway is oxycodone. Indeed, in the operant self-administration model systemic administration of a GHSR antagonist reduces the taking and breakpoint for oxycodone in male rats (81). The importance of ghrelin pathway for addictive drugs is also transferred to other addictive drugs, like cannabinoids. Indeed, GHSR antagonism attenuates whereas ghrelin enhances cannabinoid self-administration, seeking, and reward (109, 110). Collectively, these findings report that the ghrelin pathway modulate drug-taking and reward associated with additive drugs in male rodents.

## 3. Confounding factors

Although the above-mentioned articles provide support for that alcohol effects (drinking, behaviors, and neurochemistry) are enhanced by ghrelin and reduced by GHSR suppression, there are confounding factors that may influence the interpretation of these data. One such may be caloric intake and consummatory behaviors as these are well-established functions of ghrelin [for review see Espinoza Garcia et al. (111)]. This appears less likely as GHSR antagonism prevents alcohol responses like hyperlocomotion, dopamine release in NAc and CPP that are less controlled by such factors [for review see Shevchouk et al. (12)]. Yet another support for that reward, rather than consummatory behaviors and calories, are the findings that the ghrelin pathway controls drug responses which are driven by reward rather than consumption/calories. On a final note, in a choice situation GHSR antagonism decreases the most rewarding food, suggesting that reward rather than general consumption is affected. When using GHSR antagonists like [D-Lys^3^]-GHRP-6 receptor unselectively may be a concern. However, this appears less likely to influence the interpretation as different antagonists display similar results and opposite results to ghrelin itself. The possibility that enhanced alcohol metabolism rather than reduced alcohol reward should be taken into consideration as another confounding factor. Although not studied for the ghrelin pathway, gut-brain peptides like GLP-1 and amylin do not display such effect (112, 113). Stress is a factor that could influence the ability of the ghrelin pathway to control alcohol effects as ghrelin effects the hypothalamus-pituitary-adrenal axis (114, 115). When it comes to GLP-1, nausea/aversion is a common side effect that might influence the obtained data. This may also a possibility when it comes to ghrelin pathway as ghrelin decreases nausea (116). However, this may be less likely as GHSR antagonists do not (i) reduce water intake, (ii) alter CPP alone, or (iii) changes locomotion or NAC-dopamine *per se*.

## 4. Future directions and concluding remarks

Although these preclinical studies show that different GHSR antagonists have a similar ability to decline alcohol drinking, all but one study (25) are conducted in male animals. Besides, the neurobiological underpinnings of AUD may diverge between sexes [for review see Becker et al. (117)], and a sex-divergent difference is also when it comes to ghrelin in plasma from AUD patients (38). Upcoming studies should thus evaluate the influence of the ghrelin pathway on alcohol-mediated behaviors in female subjects. Although the above reviewed studies imply that reward-related areas are central for the alcohol-ghrelin interaction, additional studies are warranted in an attempt to define brain nodes, circuits and neurobiological substrate central for this interaction. One of the mechanisms that should be explored in detail is the ghrelin-dopamine-VTA interaction that has been implied in separate studies (7, 68, 70–72). Moreover, reward has been suggested as a central mechanism contributing to the ability of the ghrelin pathway to control alcohol drinking, but other tentative biological mechanisms should be evaluated for this interaction. Therefore future studies should explore how ghrelin affects impulsivity and risk-taking, behaviors central for AUD diagnosis. Although human and rodent studies reveal similar expression of GHSR (55–57, 64), differences are evident when it comes to effects and regions involved (17, 64). Therefore, studies should explore if similar or diverging mechanisms contribute to the ghrelin-alcohol association in humans as in rodents. Importantly, such studies should control for factors like time of alcohol exposure, withdrawal or not, gender and feeding status. Although available research indicates that GHSR are required for several steps of the AUD cycle, its role for drinking to avoid negative consequences are unknown and a subject for upcoming studies. When it comes to feeding and body weight, the combination of gut-brain peptides synergistic-like decline these parameters (118). Therefore, upcoming studies should test the possibility that the combination of a GHSR antagonist with other gut-brain peptides synergistically or additively reduces on alcohol drinking.

As reviewed above, ghrelin and its receptor appear central for alcohol responses. However, other substrates of the ghrelin pathway exist and their role in addiction processes is unknown. The ghrelin gene is translated into ghrelin-obestatin, which is converted to pro-ghrelin which after acylation by GOAT and conversion forms the active form of ghrelin; acyl-ghrelin (here denominated ghrelin) [for review see Muller et al. (119)]. Ghrelin is then formed into des-acyl-ghrelin (DAG) (120), which previously was considered inactive. However, recent advances show that DAG affects behaviors as it regulates feeding (121) and block physiological properties of ghrelin (121–123). Although, plasma levels of DAG are not associated with alcohol craving in humans (40, 41) its association with various aspects of the AUD process should be elucidated in detail. Another substrate of the ghrelin pathway is LEAP2, an endogenous inverse GHSR agonist that appears to reduce ghrelin-induced behaviors and impair dopaminergic signaling (72, 124–126). As the role of GOAT, DAG, or LEAP2 in addiction processes is unknown, future studies should explore this in detail; both in humans and animals. Besides, LEAP2 has not been explored in relation to alcohol craving or other aspects of the AUD cycle, a tentative focus on warranted plasma-association studies. Speculatively, these studies could be used to identify individuals with elevated alcohol craving that tentatively could benefit from agents targeting the ghrelin pathway. Another aspect that needs further attention is the role of central versus peripheral ghrelin signaling in addiction processes.

In conclusion, the vast number of preclinical studies reveal that the ghrelin pathway is central for reward, reinforcement and addiction processes. Besides, findings from these preclinical studies are accompanied with supporting studies in humans [for review see Morris et al. (127)]. When identifying mechanisms like the ghrelin pathway which is central for AUD, indirect insight into impulsivity and risk-taking are provided. Collectively these studies indicate that pharmacological approaches to dampen the GHSR should be evaluated in patients with AUD.

## Author contributions

EJ conducted the literature search for the present review article, summarized the work conducted within the field, and wrote the review.
